# Supplementary material for: Phosphorus-rich stars with unusual abundances are challenging theoretical predictions
Source: Nat Commun. 2020 Aug 4;11:3759. doi: 10.1038/s41467-020-17649-9 (PMC7403594; doi:10.1038/s41467-020-17649-9)
Supplement: Supplementary file 1 — Supplementary Information [file 41467_2020_17649_MOESM1_ESM.pdf]

Phosphorus-rich stars with unusual abundances are challenging theoretical predictions

Thomas Masseron, D. A. García-Hernández, Raúl Santoveña Gómez, Arturo Manchado Torres, Olga Zamora, Minia Manteiga, Carlos Dafonte

June 18, 2020

Supplementary Table 1: Basic parameters of the P-rich stars

star	T_{eff}	log g	μ_t	[Fe/H]	SNR	dist.	Lum.	<rv>	rms	#visit	Δt
2M00044180-0005553	4597(33)	2.01(0.13)	1.38	$-1.01^{0.13}_{0.00}$	553	4300	276	-59.67	0.08	10	300
2M12071315+1555010	5119(29)	2.24(0.28)	1.33	$-0.87^{0.15}_{0.01}$	55	9700	264	28.49	0.05	3	411
2M13314691+2804210	4224(177)	1.22(0.52)	1.66	$-1.02^{0.14}_{0.03}$	249	9000	1120	-1.80	0.03	4	8
2M13472354+2210562	5377(295)	2.2(1.56)	1.34	$-1.24^{0.21}_{0.17}$	164	2200	60	30.75	0.47	3	3
2M13535604+4437076	5143(97)	2.5(0.82)	1.30	$-0.89^{0.19}_{0.02}$	346	1500	60	-128.88	0.01	3	346
2M16092248+2449223	4208(75)	1.07(0.1)	1.74	$-1.06^{0.2}_{0.04}$	363	9800	1561	-92.42	0.29	5	15
2M16482145-1930487	4481(57)	1.61(1.31)	1.50	$-0.87^{0.17}_{0.03}$	98	8000	330	65.93	0.04	3	706
2M17033361-2254246	4783(84)	2.27(0.03)	1.33	$-0.77^{0.35}_{0.02}$	62	8700	49	81.50	1.76^{*}	15	388
2M17183459+4302520	5130(1)	2.66(0.25)	1.29	$-0.91^{0.22}_{0.00}$	91	10900	92	-135.80	0.27	15	371
2M17214096+4246147	4550(67)	1.49(0.06)	1.54	$-0.98^{0.14}_{0.02}$	179	9800	883	-297.56	0.07	5	31
2M17502038-2805411	3885(46)	0.28(0.54)	2.25	$-1.01^{0.15}_{0.12}$	101	4300	249	-52.73	0.13	2	11
2M18120031-1350169	4244(179)	1.36(0.1)	1.60	$-0.88^{0.15}_{0.07}$	130	5000	200	-36.24	0.00	1	0
2M19105369+2717150	5255(31)	3.21(0.14)	1.32	$-0.83^{0.2}_{0.03}$	73	4700	43	-5.08	0.18	7	11
2M19214936-1232462	4096(97)	1.09(0.46)	1.73	$-0.91^{0.14}_{0.04}$	266	12300	1277	-146.57	0.23	3	222
2M19281906+4915086	4425(109)	1.63(0.15)	1.49	$-1.15^{0.13}_{0.05}$	374	4700	603	-302.63	0.21	2	50

Effective temperatures (T_{eff} in K) and surface gravities ($\log g$) uncertainties are shown in parentheses. μ_t are the microturbulence velocities (in km s^{-1}) assumed for the abundances derivation. [Fe/H] represent the metallicities of the stars relative to the solar value based on Fe lines measurement. The upper indices show the random errors while the bottom indices show the systematic errors. dist are the distances of the stars in parsec. Lum. are the stars luminosities relative to the Solar one. $< \mathrm{rv} >$ show the average radial velocities, rms the dispersion around that value (both in km s^{-1}) and #visit the number of observed spectra. and Δt (in days) the total observation period range.

* star suspected to be a binary

Supplementary Table 2: Basic parameters of the P-normal stars

star	T_{eff}	$\log$ g	μ_t	[Fe/H]	SNR	dist.	Lum.	<rv>	rms	#visit
2M00022364+1558539	5229(79)	2.4(0.88)	1.31	$-0.77^{0.13}_{0.01}$	301	1900	57	-60.30	0.05	3
2M01262518+1719099	5232(130)	2.39(1.03)	1.31	$-0.84^{0.11}_{0.01}$	724	1000	55	35.53	0.21	5
2M01271266+1801037	4730(216)	2.17(0.21)	1.35	$-1.13^{0.13}_{0.1}$	1405	700	96	101.02	0.53	5
2M02150319-0437411	5027(177)	2.9(0.08)	1.29	$-0.97^{0.16}_{0.1}$	550	700	20	80.77	0.10	3
2M07404920+3621206	4610(54)	2.26(0.28)	1.33	$-0.65^{0.13}_{0.00}$	358	2900	71	66.74	0.04	7
2M07461742+4828201	4709(182)	2.03(0.25)	1.37	$-1.05^{0.24}_{0.11}$	410	2400	194	-39.46	0.07	2
2M11202609+0024314	4391(94)	1.02(0.47)	1.76	$-1.1^{0.15}_{0.05}$	271	9800	846	195.36	0.30	3
2M13240275+2516183	5168(138)	2.29(0.99)	1.33	$-0.86^{0.14}_{0.04}$	305	1300	69	69.02	0.04	3
2M15563661+2716483	4533(165)	1.78(0.1)	1.44	$-1.01^{0.18}_{0.02}$	327	2700	315	17.36	0.01	2
2M16164586+4652131	4668(113)	1.97(0.07)	1.39	$-1.01^{0.14}_{0.06}$	305	2500	140	-112.77	0.04	3
2M16471103-0156177	4566(640)	1.74(1.26)	1.45	$-0.99^{0.17}_{0.29}$	264	8700	2441	-44.43	0.00	1
2M17142525+4324562	4490(110)	1.7(0)	1.47	$-1.04^{0.14}_{0.05}$	406	5700	341	-106.93	0.04	5
2M19311218-0840354	4765(27)	2.09(0.02)	1.36	$-0.65^{0.14}_{0.01}$	212	5200	211	18.51	0.28	3
2M19320870+4926497	4733(123)	2.4(0.21)	1.31	$-0.74^{0.09}_{0.06}$	212	1600	54	-80.09	0.00	1
2M21330531+0041464	4548(42)	2.05(0.19)	1.37	$-0.82^{0.13}_{0.03}$	476	1900	120	-96.13	0.12	6

Effective temperatures (T_{eff} in K) and surface gravities ($\log g$) uncertainties are shown in parentheses. μ_t are the microturbulence velocities (in km s^{-1}) assumed for the abundances derivation. $[Fe/H]$ represent the metallicities of the stars relative to the solar value based on Fe lines measurement. The upper indices show the random errors while the bottom indices show the systematic errors. dist are the distances of the stars in parsec. $\mathrm{Lum.}$ are the stars luminosities relative to the Solar one. $< \mathrm{rv} >$ show the average radial velocities, rms the dispersion around that value (both in km s^{-1}) and $\#\mathrm{visit}$ the number of observed spectra.

Supplementary Table 3: Abundances of the P-rich stars

star	[C/Fe]	[N/Fe]	[O/Fe]	[Na/Fe]	[Mg/Fe]	[Al/Fe]	[Si/Fe]	[P/Fe]	[S/Fe]
2M00044180	$0.24^{0.05}_{0.08}$	$0.35^{0.14}_{0.09}$	$0.71^{0.03}_{0.07}$	$<0.42^{\cdots}_{\cdots}$	$0.47^{0.1}_{0.04}$	$0.7^{0.02}_{0.09}$	$0.59^{0.06}_{0.02}$	$1.35^{0.3}_{0.09}$	$0.32^{0.04}_{0.08}$
2M12071315	$0.06^{0.06}_{0.39}$	$<0.91^{\cdots}_{\cdots}$	$<1.25^{\cdots}_{\cdots}$	$<0.27^{\cdots}_{\cdots}$	$0.71^{0.09}_{0.09}$	$1.3^{0.1}_{0.09}$	$0.9^{0.13}_{0.04}$	$1.2^{0.16}_{0.1}$	$0.42^{0.14}_{0.11}$
2M13314691	$-0.06^{0.17}_{0.16}$	$0.77^{0.05}_{0.19}$	$0.89^{0.08}_{0.29}$	$<0.31^{\cdots}_{\cdots}$	$0.58^{0.09}_{0.05}$	$1.11^{0.01}_{0.09}$	$0.86^{0.12}_{0.16}$	$1.53^{0.31}_{0.1}$	$0.43^{0.12}_{0.25}$
2M13472354	$0.33^{0.04}_{0.09}$	$<1.46^{\cdots}_{\cdots}$	...$^{\cdots}_{\cdots}$	$<0.71^{\cdots}_{\cdots}$	$0.59^{0.11}_{0.3}$	$0.86^{0.03}_{0.09}$	$0.87^{0.12}_{0.24}$	$1.65^{0.26}_{0.1}$	$0.56^{0.13}_{0.1}$
2M13535604	$-0.06^{0.02}_{0.68}$	$<0.77^{\cdots}_{\cdots}$	$<1.56^{\cdots}_{\cdots}$	$<0.28^{\cdots}_{\cdots}$	$0.91^{0.1}_{0.24}$	$1.75^{0.01}_{0.09}$	$1.39^{0.13}_{0.09}$	$2.1^{0.25}_{0.1}$	$0.5^{0.13}_{0.09}$
2M16092248	$-0.05^{0.11}_{0.13}$	$<0.83^{\cdots}_{\cdots}$	$1.17^{0.06}_{0.29}$	$<0.35^{\cdots}_{\cdots}$	$0.68^{0.1}_{0.01}$	$1.29^{0}_{0.1}$	$1.02^{0.13}_{0.02}$	$1.81^{0.21}_{0.09}$	$0.12^{0.19}_{0.2}$
2M16482145	$0.11^{0.14}_{0.35}$	$<0.41^{\cdots}_{\cdots}$	$0.89^{0.05}_{0.11}$	$<0.23^{\cdots}_{\cdots}$	$0.67^{0.15}_{0.04}$	$1.19^{0.07}_{0.09}$	$0.79^{0.14}_{0.16}$	$1.57^{0.17}_{0.1}$	$0.41^{0.21}_{0.46}$
2M17033361	$0.06^{0.08}_{0.12}$	$0.41^{0.2}_{0.09}$	$<0.71^{\cdots}_{\cdots}$	$<0.14^{\cdots}_{\cdots}$	$0.68^{0.12}_{0.01}$	$1.22^{0.06}_{0.1}$	$0.73^{0.14}_{0.02}$	$1.8^{0.31}_{0.09}$	$0.03^{0.2}_{0.1}$
2M17183459	$0.07^{0.19}_{0.02}$	$<1.17^{\cdots}_{\cdots}$	$<1.21^{\cdots}_{\cdots}$	$<0.37^{\cdots}_{\cdots}$	$0.67^{0.08}_{0.04}$	$1.48^{0.01}_{0.1}$	$1^{0.14}_{0.01}$	$1.37^{0.03}_{0.09}$	$0.34^{0.01}_{0.03}$
2M17214096	$-0.11^{0.14}_{0.04}$	$<0.72^{\cdots}_{\cdots}$	$1^{0.06}_{0.04}$	$<0.48^{\cdots}_{\cdots}$	$0.64^{0.14}_{0.01}$	$1.32^{0.04}_{0.09}$	$1.02^{0.12}_{0.04}$	$2.04^{0.02}_{0.1}$	$0.44^{0.09}_{0.11}$
2M17502038	$-0.12^{0.09}_{0.09}$	$<0.61^{\cdots}_{\cdots}$	$1.16^{0.07}_{0.1}$	$<0.16^{\cdots}_{\cdots}$	$0.7^{0.15}_{0.1}$	$1.42^{0.17}_{0.1}$	$0.75^{0.11}_{0}$	$1.69^{0.1}_{0.1}$	$0.27^{0.2}_{0.1}$
2M18120031	$0.01^{0.12}_{0.1}$	$0.97^{0.07}_{0.47}$	$0.96^{0.04}_{0.1}$	$<0.12^{\cdots}_{\cdots}$	$0.57^{0.12}_{0.05}$	$1.22^{0.03}_{0.1}$	$0.76^{0.12}_{0}$	$1.65^{0.08}_{0.1}$	$0.35^{0.16}_{0.17}$
2M19105369	$0.04^{0.2}_{0.84}$	$1.42^{0.19}_{0.64}$	$<0.97^{\cdots}_{\cdots}$	$<0.27^{\cdots}_{\cdots}$	$0.61^{0.11}_{0.05}$	$1.1^{0.01}_{0.09}$	$0.87^{0.11}_{0.03}$	$1.69^{0.23}_{0.1}$	$0.49^{0.2}_{0.05}$
2M19214936	$0.05^{0.05}_{0.07}$	$0.6^{0.12}_{0.05}$	$0.91^{0.05}_{0.12}$	$0.12^{0.13}_{0.1}$	$0.53^{0.08}_{0.05}$	$0.93^{0.01}_{0.09}$	$0.71^{0.12}_{0.09}$	$1.44^{0.24}_{0.1}$	$0.21^{0.07}_{0.19}$
2M19281906	$-0.11^{0.11}_{0.08}$	$<0.7^{\cdots}_{\cdots}$	$0.73^{0.05}_{0.13}$	$<0.56^{\cdots}_{\cdots}$	$0.47^{0.13}_{0.02}$	$0.89^{0.05}_{0.09}$	$0.72^{0.11}_{0.03}$	$1.28^{0.07}_{0.1}$	$0.24^{0.07}_{0}$
star	[K/Fe]	[Ca/Fe]	[Ti/Fe]	[V/Fe]	[Cr/Fe]	[Mn/Fe]	[Co/Fe]	[Ni/Fe]	[Ce/Fe]
2M00044180	$0.08^{0.03}_{0.01}$	$0.27^{0.02}_{0.03}$	$0.24^{0.03}_{0.01}$	$<0.72^{\cdots}_{\cdots}$	$<0.22^{\cdots}_{\cdots}$	$-0.42^{0.05}_{0.01}$	$<0.15^{\cdots}_{\cdots}$	$0.11^{0.07}_{0.06}$	$<0.02^{\cdots}_{\cdots}$
2M12071315	$0.35^{0.02}_{0.27}$	$0.19^{0.08}_{0.01}$	$0.25^{0.01}_{0.14}$	$<1.78^{\cdots}_{\cdots}$	$<-0.06^{\cdots}_{\cdots}$	$-0.2^{0.11}_{0.1}$	$<0.43^{\cdots}_{\cdots}$	$0.06^{0.13}_{0}$	$0.67^{0.09}_{0.33}$
2M13314691	$0.06^{0.06}_{0.09}$	$0.31^{0.02}_{0.09}$	$0.4^{0.1}_{0.26}$	$0.23^{0.04}_{0.1}$	$0.04^{0.05}_{0.01}$	$-0.33^{0.04}_{0}$	$0.09^{0.11}_{0.02}$	$0.09^{0.05}_{0.03}$	$0.79^{0.08}_{0.17}$
2M13472354	$<0.36^{\cdots}_{\cdots}$	$0.12^{0.05}_{0.08}$	$<0.58^{\cdots}_{\cdots}$	...$^{\cdots}_{\cdots}$	$<0.56^{\cdots}_{\cdots}$	$<0.19^{\cdots}_{\cdots}$	$<1.47^{\cdots}_{\cdots}$	$0.18^{0.1}_{0.1}$	$<0.77^{\cdots}_{\cdots}$
2M13535604	$0.16^{0.03}_{0.19}$	$0.28^{0.1}_{0.06}$	$0.16^{0.07}_{0.1}$	$<0.89^{\cdots}_{\cdots}$	$<0.25^{\cdots}_{\cdots}$	$-0.06^{0.24}_{0.19}$	$<0.51^{\cdots}_{\cdots}$	$0.28^{0.12}_{0.01}$	$0.85^{0.1}_{0.09}$
2M16092248	$0.09^{0}_{0.04}$	$0.15^{0.07}_{0.01}$	$0.32^{0.2}_{0.05}$	$<0.17^{\cdots}_{\cdots}$	$<-0.07^{\cdots}_{\cdots}$	$<-0.45^{\cdots}_{\cdots}$	$0.21^{0.08}_{0.04}$	$0.2^{0.05}_{0}$	$0.78^{0.11}_{0.09}$
2M16482145	$0.12^{0.11}_{0.01}$	$0.25^{0.05}_{0.07}$	$0.43^{0.16}_{0.09}$	$<0.45^{\cdots}_{\cdots}$	$<0.03^{\cdots}_{\cdots}$	$-0.11^{0.27}_{0.01}$	$0.04^{0.12}_{0}$	$0.02^{0.05}_{0.1}$	$0.68^{0.09}_{0.58}$
2M17033361	$0.17^{0.01}_{0.11}$	$0.38^{0.13}_{0.01}$	$0.7^{0.21}_{0.08}$	$<0.71^{\cdots}_{\cdots}$	$<-0.16^{\cdots}_{\cdots}$	$0.00^{0.25}_{0.19}$	$0.28^{0.15}_{0.04}$	$0.02^{0.24}_{0}$	$1.04^{0.14}_{0.04}$
2M17183459	$0.06^{0.05}_{0.06}$	$0.27^{0.1}_{0}$	$0.77^{0.15}_{0.07}$	$<1.23^{\cdots}_{\cdots}$	$<0.27^{\cdots}_{\cdots}$	$-0.18^{0.13}_{0.02}$	$<0.48^{\cdots}_{\cdots}$	$0.07^{0.12}_{0}$	$0.68^{0.23}_{0.3}$
2M17214096	$0.16^{0.03}_{0.07}$	$0.04^{0.11}_{0.08}$	$0.32^{0.1}_{0.1}$	$<0.63^{\cdots}_{\cdots}$	$<0.14^{\cdots}_{\cdots}$	$-0.26^{0.05}_{0.03}$	$0.19^{0.02}_{0.01}$	$0.1^{0.05}_{0.04}$	$0.79^{0.1}_{0.09}$
2M17502038	$0.38^{0.06}_{0.08}$	$0.48^{0.03}_{0.08}$	$0.49^{0.15}_{0.06}$	$0.43^{0.06}_{0.03}$	$0.12^{0.05}_{0.24}$	$-0.28^{0.07}_{0.06}$	$0.06^{0.04}_{0.02}$	$-0.19^{0.19}_{0.27}$	$0.57^{0.15}_{0.1}$
2M18120031	$0.21^{0.02}_{0.02}$	$0.34^{0.02}_{0.06}$	$0.45^{0.14}_{0.25}$	$0.28^{0.07}_{0.1}$	$<0.14^{\cdots}_{\cdots}$	$-0.21^{0.3}_{0.1}$	$0.07^{0.06}_{0.1}$	$-0.03^{0.11}_{0.12}$	$0.74^{0.17}_{0.11}$
2M19105369	$0.26^{0.05}_{0.04}$	$0.23^{0.15}_{0.07}$	$0.55^{0.2}_{0.02}$	$<2.06^{\cdots}_{\cdots}$	$<0.19^{\cdots}_{\cdots}$	$-0.1^{0.12}_{0.01}$	$<0.5^{\cdots}_{\cdots}$	$0.1^{0.13}_{0}$	$1.11^{0.05}_{0.09}$
2M19214936	$0.11^{0.01}_{0.12}$	$0.31^{0.01}_{0.13}$	$0.36^{0.19}_{0.14}$	$0.17^{0.07}_{0.12}$	$-0.09^{0.08}_{0.27}$	$-0.23^{0.2}_{0.05}$	$0.16^{0.02}_{0.03}$	$-0.01^{0.06}_{0.04}$	$0.7^{0.13}_{0.15}$
2M19281906	$0.13^{0.04}_{0.07}$	$0.19^{0.02}_{0.01}$	$0.38^{0.2}_{0.1}$	$<0.63^{\cdots}_{\cdots}$	$-0.03^{0.27}_{0.08}$	$-0.05^{0.29}_{0.1}$	$0.13^{0.06}_{0.03}$	$0.15^{0.04}_{0.02}$	$0.75^{0.13}_{0.22}$

All abundances are in logarithmic scale, relative to their metallicity and to the Solar values[1] such as displayed in Fig.1, 4 and 6 . The upper indices show the random errors while the bottom indices show the systematic errors.

Supplementary Table 4: Abundances of the P-normal stars

star	[C/Fe]	[N/Fe]	[O/Fe]	[Na/Fe]	[Mg/Fe]	[Al/Fe]	[Si/Fe]	[P/Fe]	[S/Fe]
2M00022364	$0.24^{0.19}_{0.13}$	$0.38^{0.07}_{0.1}$	$<0.56^{\cdots}_{\cdots}$	$<0.29^{\cdots}_{\cdots}$	$0.43^{0.08}_{0.11}$	$0.45^{0.12}_{0.17}$	$0.37^{0.09}_{0.05}$	$<0.51^{\cdots}_{\cdots}$	$0.26^{0.17}_{0.14}$
2M01262518	$0.29^{0.18}_{0.02}$	$0.2^{0.03}_{0.1}$	$0.52^{0.17}_{0.1}$	$<0.47^{\cdots}_{\cdots}$	$0.4^{0.07}_{0.08}$	$0.45^{0.09}_{0.14}$	$0.34^{0.09}_{0.02}$	$<0.32^{\cdots}_{\cdots}$	$0.27^{0.17}_{0.05}$
2M01271266	$0.09^{0.06}_{0.15}$	$0.17^{0.03}_{0.06}$	$0.61^{0.11}_{0.09}$	$0.02^{0.18}_{0.1}$	$0.4^{0.11}_{0.03}$	$0.36^{0.02}_{0.02}$	$0.3^{0.07}_{0.03}$	$0.41^{0.28}_{0.09}$	$0.34^{0.17}_{0.1}$
2M02150319	$0.32^{0.03}_{0.23}$	$0.23^{0.22}_{0.48}$	$0.61^{0.05}_{0.09}$	$0.46^{0.19}_{0.1}$	$0.41^{0.09}_{0.04}$	$0.37^{0.00}_{0.00}$	$0.25^{0.07}_{0.04}$	$<-0.25^{\cdots}_{\cdots}$	$0.18^{0.11}_{0.1}$
2M07404920	$0.13^{0.09}_{0.12}$	$0.19^{0.08}_{0.15}$	$0.54^{0.04}_{0.11}$	$0.09^{0.14}_{0.04}$	$0.34^{0.13}_{0.1}$	$0.44^{0.03}_{0.09}$	$0.3^{0.07}_{0.03}$	$<0.67^{\cdots}_{\cdots}$	$0.27^{0.08}_{0.16}$
2M07461742	$0.22^{0.13}_{0.06}$	$0.24^{0.05}_{0.06}$	$0.6^{0.03}_{0.04}$	$<0.38^{\cdots}_{\cdots}$	$0.33^{0.05}_{0.06}$	$0.36^{0.05}_{0.00}$	$0.24^{0.06}_{0.03}$	$<0.66^{\cdots}_{\cdots}$	$0.14^{0.1}_{0.03}$
2M11202609	$0.29^{0.03}_{0.15}$	$0.46^{0.04}_{0.01}$	$0.5^{0.05}_{0.01}$	$0.53^{0.12}_{0.1}$	$0.4^{0.13}_{0.04}$	$-0.02^{0.03}_{0.04}$	$0.25^{0.1}_{0.08}$	$<0.59^{\cdots}_{\cdots}$	$0.45^{0.2}_{0.06}$
2M13240275	$0.15^{0.24}_{0.21}$	$0.21^{0.15}_{0.00}$	$<0.46^{\cdots}_{\cdots}$	$<0.39^{\cdots}_{\cdots}$	$0.41^{0.1}_{0.08}$	$0.38^{0.11}_{0.11}$	$0.33^{0.1}_{0.06}$	$0.33^{0.33}_{0.09}$	$0.32^{0.25}_{0.06}$
2M15563661	$0.04^{0.08}_{0.00}$	$0.29^{0.03}_{0.05}$	$0.56^{0.13}_{0.03}$	$0.28^{0.25}_{0.1}$	$0.4^{0.08}_{0.09}$	$0.45^{0.05}_{0.05}$	$0.29^{0.07}_{0.00}$	$<0.11^{\cdots}_{\cdots}$	$0.18^{0.02}_{0.07}$
2M16164586	$0.00^{0.11}_{0.09}$	$0.18^{0.04}_{0.06}$	$0.59^{0.11}_{0.04}$	$<0.29^{\cdots}_{\cdots}$	$0.42^{0.08}_{0.00}$	$0.38^{0.00}_{0.02}$	$0.32^{0.06}_{0.01}$	$\ldots^{\cdots}_{\cdots}$	$0.26^{0.06}_{0.04}$
2M16471103	$-0.39^{0.12}_{0.05}$	$1.48^{0.03}_{0.1}$	$0.53^{0.1}_{0.55}$	$0.39^{0.12}_{0.09}$	$0.35^{0.08}_{0.2}$	$0.44^{0.26}_{0.03}$	$0.26^{0.09}_{0.06}$	$\ldots^{\cdots}_{\cdots}$	$0.11^{0.07}_{0.08}$
2M17142525	$-0.2^{0.14}_{0.00}$	$0.2^{0.04}_{0.11}$	$0.55^{0.06}_{0.1}$	$0.22^{0.17}_{0.1}$	$0.31^{0.1}_{0.00}$	$0.01^{0.09}_{0.02}$	$0.25^{0.07}_{0.04}$	$<0.58^{\cdots}_{\cdots}$	$0.25^{0.15}_{0.15}$
2M19311218	$0.15^{0.06}_{0.00}$	$0.38^{0.07}_{0.02}$	$0.6^{0.08}_{0.03}$	$<-0.02^{\cdots}_{\cdots}$	$0.42^{0.1}_{0.00}$	$0.55^{0.02}_{0.00}$	$0.39^{0.09}_{0.00}$	$0.47^{0.2}_{0.1}$	$0.33^{0.11}_{0.02}$
2M19320870	$0.16^{0.06}_{0.06}$	$<0.18^{\cdots}_{\cdots}$	$0.62^{0.08}_{0.14}$	$<0.1^{\cdots}_{\cdots}$	$0.39^{0.11}_{0.02}$	$0.5^{0.04}_{0.1}$	$0.29^{0.08}_{0.04}$	$0.78^{0.2}_{0.09}$	$0.33^{0.1}_{0.15}$
2M21330531	$0.09^{0.06}_{0.13}$	$<0.25^{\cdots}_{\cdots}$	$0.65^{0.06}_{0.01}$	$<0.16^{\cdots}_{\cdots}$	$0.4^{0.1}_{0.08}$	$0.54^{0.03}_{0.1}$	$0.44^{0.06}_{0.01}$	$<0.84^{\cdots}_{\cdots}$	$0.27^{0.07}_{0.11}$

star	[K/Fe]	[Ca/Fe]	[Ti/Fe]	[V/Fe]	[Cr/Fe]	[Mn/Fe]	[Co/Fe]	[Ni/Fe]	[Ce/Fe]
2M00022364	$0.19^{0.00}_{0.04}$	$0.15^{0.04}_{0.03}$	$0.14^{0.07}_{0.74}$	$\ldots^{\cdots}_{\cdots}$	$-0.2^{0.1}_{0.1}$	$-0.41^{0.05}_{0.06}$	$<-0.1^{\cdots}_{\cdots}$	$0.03^{0.12}_{0.11}$	$0.25^{0.32}_{0.1}$
2M01262518	$0.06^{0.07}_{0.16}$	$0.06^{0.01}_{0.00}$	$0.09^{0.2}_{0.86}$	$<1.8^{\cdots}_{\cdots}$	$-0.28^{0.2}_{0.1}$	$-0.28^{0.25}_{0.1}$	$<0.12^{\cdots}_{\cdots}$	$0.05^{0.04}_{0.11}$	$<0.11^{\cdots}_{\cdots}$
2M01271266	$0.15^{0.05}_{0.28}$	$0.3^{0.04}_{0.00}$	$0.38^{0.2}_{0.06}$	$<-0.88^{\cdots}_{\cdots}$	$-0.08^{0.00}_{0.1}$	$-0.37^{0.19}_{0.12}$	$0.13^{0.18}_{0.1}$	$0.05^{0.04}_{0.04}$	$0.24^{0.1}_{0.11}$
2M02150319	$0.14^{0.06}_{0.06}$	$0.17^{0.01}_{0.06}$	$0.11^{0.09}_{0.05}$	$<0.34^{\cdots}_{\cdots}$	$0.1^{0.00}_{0.1}$	$-0.32^{0.11}_{0.04}$	$0.14^{0.18}_{0.1}$	$0.18^{0.08}_{0.01}$	$<-0.2^{\cdots}_{\cdots}$
2M07404920	$0.18^{0.03}_{0.03}$	$0.16^{0.02}_{0.05}$	$0.29^{0.16}_{0.13}$	$<0.36^{\cdots}_{\cdots}$	$-0.13^{0.06}_{0.01}$	$-0.34^{0.03}_{0.14}$	$0.07^{0.12}_{0.01}$	$0.07^{0.00}_{0.06}$	$<-0.18^{\cdots}_{\cdots}$
2M07461742	$0.18^{0.07}_{0.38}$	$0.22^{0.05}_{0.02}$	$0.12^{0.14}_{0.1}$	$<0.55^{\cdots}_{\cdots}$	$0.03^{0.06}_{0.36}$	$-0.18^{0.05}_{0.1}$	$0.13^{0.1}_{0.1}$	$0.13^{0.05}_{0.06}$	$0.1^{0.14}_{0.21}$
2M11202609	$0.27^{0.00}_{0.16}$	$0.26^{0.02}_{0.05}$	$0.63^{0.23}_{0.44}$	$<-0.01^{\cdots}_{\cdots}$	$0.00^{0.07}_{0.1}$	$0.2^{0.39}_{0.46}$	$-0.13^{0.08}_{0.18}$	$0.05^{0.03}_{0.02}$	$0.94^{0.15}_{0.09}$
2M13240275	$0.11^{0.00}_{0.26}$	$0.12^{0.01}_{0.05}$	$0.2^{0.15}_{0.76}$	$<0.67^{\cdots}_{\cdots}$	$-0.07^{0.11}_{0.1}$	$-0.37^{0.07}_{0.07}$	$<-0.1^{\cdots}_{\cdots}$	$0.08^{0.07}_{0.1}$	$0.02^{0.13}_{0.1}$
2M15563661	$0.09^{0.04}_{0.08}$	$0.22^{0.03}_{0.06}$	$0.37^{0.28}_{0.09}$	$<-0.08^{\cdots}_{\cdots}$	$-0.08^{0.24}_{0.28}$	$-0.35^{0.00}_{0.08}$	$0.11^{0.12}_{0.05}$	$0.03^{0.05}_{0.04}$	$0.13^{0.06}_{0.13}$
2M16164586	$0.14^{0.05}_{0.23}$	$0.28^{0.00}_{0.00}$	$0.15^{0.07}_{0.07}$	$0.24^{0.11}_{0.1}$	$0.14^{0.04}_{0.1}$	$-0.34^{0.07}_{0.02}$	$-0.28^{0.15}_{0.1}$	$0.05^{0.03}_{0.04}$	$0.41^{0.03}_{0.03}$
2M16471103	$0.16^{0.01}_{0.3}$	$0.23^{0.03}_{0.12}$	$0.56^{0.19}_{0.67}$	$<0.16^{\cdots}_{\cdots}$	$0.14^{0.13}_{0.1}$	$-0.32^{0.01}_{0.34}$	$-0.27^{0.09}_{0.1}$	$0.04^{0.04}_{0.08}$	$0.37^{0.02}_{0.09}$
2M17142525	$0.11^{0.02}_{0.05}$	$0.23^{0.01}_{0.01}$	$0.11^{0.06}_{0.42}$	$<3.11^{\cdots}_{\cdots}$	$0.01^{0.08}_{0.03}$	$-0.2^{0.28}_{0.06}$	$-0.16^{0.13}_{0.1}$	$-0.04^{0.07}_{0.06}$	$0.17^{0.08}_{0.07}$
2M19311218	$0.12^{0.03}_{0.00}$	$0.14^{0.03}_{0.00}$	$0.47^{0.26}_{0.04}$	$<0.58^{\cdots}_{\cdots}$	$0.00^{0.16}_{0.00}$	$-0.35^{0.12}_{0.00}$	$<-0.05^{\cdots}_{\cdots}$	$0.09^{0.07}_{0.00}$	$<-0.21^{\cdots}_{\cdots}$
2M19320870	$0.09^{0.02}_{0.11}$	$0.26^{0.03}_{0.03}$	$0.37^{0.31}_{0.4}$	$<0.62^{\cdots}_{\cdots}$	$0.14^{0.09}_{0.14}$	$-0.44^{0.06}_{0.2}$	$0.17^{0.01}_{0.01}$	$0.04^{0.09}_{0.04}$	$<-0.05^{\cdots}_{\cdots}$
2M21330531	$0.05^{0.01}_{0.02}$	$0.29^{0.06}_{0.04}$	$0.18^{0.02}_{0.09}$	$<0.47^{\cdots}_{\cdots}$	$-0.06^{0.18}_{0.1}$	$-0.27^{0.26}_{0.2}$	$0.11^{0.06}_{0.03}$	$0.07^{0.07}_{0.01}$	$0.39^{0.07}_{0.29}$

All abundances are in logarithmic scale, relative to their metallicity and to the Solar values[1] such as displayed in Fig.1, 4 and 6. The upper indices show the random errors while the bottom indices show the systematic errors.

References

[1] Asplund, M., Grevesse, N., Sauval, A. J., Scott, P. The Chemical Composition of the Sun. Ann. Rev. Astron. Astrophys. **47**, 481-522 (2009)
